# β_3_-Adrenoceptor-mediated relaxation of rat and human urinary bladder: roles of BK_Ca_ channels and Rho kinase

**DOI:** 10.1007/s00210-015-1128-z

**Published:** 2015-05-09

**Authors:** Hana Cernecka, Kim Kersten, Harm Maarsingh, Carolina R. Elzinga, Igle Jan de Jong, Cees Korstanje, Martin C. Michel, Martina Schmidt

**Affiliations:** Department of Molecular Pharmacology, University of Groningen, Antonius Deusinglaan 1, 9713 AV Groningen, The Netherlands; Department of Urology, University Medical Center, Groningen, The Netherlands; Translational and Development Pharmacology Department, Astellas Pharma Europe BV, Leiderdorp, The Netherlands; Department of Pharmacology, Johannes Gutenberg University, Mainz, Germany; Anchored Signalling Group, Max-Delbrück Center for Molecular Medicine, Berlin, Germany

**Keywords:** BK_Ca_ channel, Human urinary bladder, Mirabegron, Rat urinary bladder, Rho-kinase

## Abstract

Previous studies suggest that the large-conductance Ca^2+^-activated K^+^ (BK_Ca_) channel and Rho-kinase play major roles in the control of urinary bladder tone. Here, we investigated their involvement in β-adrenoceptor (AR)-mediated relaxation of rat and human bladder. Concentration-response curves of isoprenaline and mirabegron-induced bladder relaxation were generated against passive tension and KCl- and carbachol-induced tone, in the absence or presence of the BK_Ca_ channel inhibitor iberiotoxin (100 nM) or the Rho-kinase inhibitor Y27,632 (1 μM). Myosin light chain (MLC) phosphorylation was studied by Western blot. In rat, iberiotoxin only slightly altered isoprenaline- and mirabegron-induced relaxation against KCl-induced tone but attenuated relaxation by both agonists against carbachol-induced tone. Y27,632 enhanced isoprenaline- or mirabegron-induced relaxation only against carbachol-induced tone. In humans, iberiotoxin slightly enhanced relaxation by both agonists against carbachol-induced pre-contraction. Y27,632 did not change isoprenaline-induced relaxation but enhanced that by mirabegron. Under passive tension, MLC phosphorylation was markedly reduced by both β-AR agonists, an effect insensitive to Y27,632. In the presence of carbachol, both β-AR agonists increased MLC phosphorylation, an effect reduced by Y27,632 only in the presence of 1 μM carbachol. These results indicate that the extent of BK_Ca_ channel and Rho-kinase involvement in relaxation induced by β-AR agonists depends on pre contractile stimulus and species.

**Electronic supplementary material:**

The online version of this article (doi:10.1007/s00210-015-1128-z) contains supplementary material, which is available to authorized users.

## Introduction

β-Adrenoceptors (ARs) mediate urinary bladder relaxation (Michel and Vrydag 2006) and have shown efficacy in experimental models of detrusor overactivity (Michel and Barendrecht 2008). Selective β_3_-AR agonists such as solabegron (Ohlstein et al. 2012) and mirabegron (Chapple et al. 2014) have shown efficacy in patients with overactive bladder syndrome (OAB), and the latter has obtained regulatory approval for this indication in Japan, USA, Canada and most EU countries.

The β-AR subtypes involved in urinary bladder relaxation differ between species: while relaxation of human detrusor smooth muscle is mediated predominantly, if not exclusively, by β_3_-AR, relaxation in rat bladder involves β_2_- and β_3_-ARs (Michel and Vrydag 2006). The degree of relaxation induced by β-AR agonists can differ depending on the stimulus used to induce pre-contraction; in several rat studies, relaxation against muscarinic agonists such as carbachol was weaker than against passive tension, KCl, bradykinin or serotonin (Longhurst and Levendusky 1999; Michel and Sand 2009; Witte et al. 2011; Cernecka et al. 2014). A similar lower potency and/or efficacy to relax carbachol-induced tone was observed in airways (Dale et al. 2014).

The canonical signalling pathway of β-ARs is stimulation of adenylyl cyclase to form cyclic adenosine monophosphate (cAMP). This results in activation of protein kinase A (PKA) which can suppress calcium-calmodulin-dependent myosin/actin interaction upon phosphorylation of myosin light chain kinase. While this could plausibly contribute to detrusor smooth muscle relaxation (Andersson and Arner 2004; Hashitani et al. 2004), studies with adenylyl cyclase or PKA inhibitors have detected only a small, if any, role for this pathway in bladder relaxation (Frazier et al. 2008). On the other hand, compelling evidence suggests that β-ARs can also stimulate large-conductance Ca^2+^-activated K^+^ (BK_Ca_) channels, e.g. in guinea pig (Petkov and Nelson 2005), rat (Hristov et al. 2008; Uchida et al. 2005), mouse (Brown et al. 2008) and human bladder (Afeli et al. 2013; Petkov 2014). Inhibition of BK_Ca_ channels with iberiotoxin in wild-type mouse and rat significantly reduced β-AR-mediated relaxation in response to isoprenaline and forskolin in KCl-contracted detrusor smooth muscle (Brown et al. 2008; Uchida et al. 2005; Frazier et al. 2005). However, even combined administration of inhibitors of the cAMP/PKA and the BK_Ca_ pathway inhibited β-AR-mediated bladder relaxation by less than half (Frazier et al. 2005), suggesting that other pathways might be involved (Birder et al. 2002). However, the identity and functional collaboration of these pathways is poorly understood; moreover, with regard to human bladder relaxation, even the roles of cAMP/PKA and BK_Ca_ have only been poorly characterized.

The contractile response in the normal human bladder is primarily mediated by M_3_ receptors (Hegde 2006). While M_3_ receptors in the bladder, similar to those in other tissues, couple to activation of a phospholipase C and formation of inositol phosphates (Kories et al. 2003), studies in rat, mouse and human bladder have shown that phospholipase C activation contributes only little to M_3_-receptor-mediated bladder contraction (Frazier et al. 2008). In contrast, entry of extracellular calcium through L-type Ca^2+^ channels and activation of Rho-kinase appear to play important roles in rat and human bladder contraction (Fleichman et al. 2004; Schneider et al. 2004a, b). Moreover, activation of Rho-kinase is suggested to play a role in several conditions associated with detrusor overactivity (Peters et al. 2006). Rho-kinase signals via a pathway encompassing myosin light chains (MLCs), CPI-17, and inhibition of MLC phosphatase (Fukata et al. 2001) resulting in an increase in myofilament calcium sensitivity and MLC phosphorylation (Somlyo and Somlyo 2003).

Therefore, we have examined the role of BK_Ca_ channels in relaxation mediated by the β_3_-AR agonist mirabegron against KCl-induced contraction in rat urinary bladder and carbachol-induced contraction in both rat and human urinary bladder. Moreover, we have studied whether inhibition of Rho-kinase might not only attenuate effects of contractile stimuli (Schneider et al. 2005), but rather also modulate β-AR-induced relaxation. Importantly, evidence is provided that the relaxing properties of mirabegron may involve change of phosphorylation status of MLC. Isoprenaline, which similarly activates all subtypes of β-AR, was used as a reference agonist in all assays.

## Materials and methods

### Tissue preparation

Human detrusor tissue was obtained from macroscopically tumour-free parts of the bladder of patients (20 males and 8 females; age range 42–79 years old) undergoing cystectomy for bladder cancer from at the Department of Urology, University Medical Center Groningen (The Netherlands) using anonymously coded, wasted tissue according to the Dutch Code of Conduct for Responsible Use (www.federa.org; one patient received neoadjuvant cytotoxic chemotherapy and some patients received BCG instalations). Tissues were placed in ice-cold Krebs–Henseleit solution (KH solution, composition in mM: 119 NaCl, 4.7 KCl, 1.2 MgSO_4_, 0.027 Na_4_EDTA, 2.5 CaCl_2_, 1.2 KH_2_PO_4_, 25 NaHCO_3_, 5.5 glucose, 10 HEPES) and transported to the laboratory immediately after surgery. Bladder strips (approximate diameter 2 mm, length 15 mm, weight 21 mg) were prepared and stored in ice-cold KH solution until next day, when the experiment was performed. We have previously shown that such storage does not affect contraction or relaxation responses (Schneider et al. 2011).

All animal experiments were approved by the University of Groningen Committee for Animal Experimentation. Male Wistar rats (*n* = 47, 250–300 g) obtained from Harlan (Horst, The Netherlands) were housed 7 days before experiments with free access to food and water. Animals were anaesthetized with CO_2_ and sacrificed by exsanguination. The bladder was removed, cleaned of connective tissue and cut into strips (approximate diameter 1 mm, length 20 mm, weight 10 mg). No attempts were made to remove urothelium during strip preparation in rats or humans, as urothelium removal had not affected contractile responses to carbachol in rat bladder in our previous studies (Michel 2014).

### Organ bath studies

Studies with rat and human bladder strips were adapted from a protocol previously described for airway strips (Boterman et al. 2005), with the following modifications. Tissue strips were mounted under a tension of 10 mN in 20 mL organ baths containing KH solution, which was kept at 37 °C and aerated with 95 % O_2_ and 5 % CO_2_ to maintain pH 7.4. Bladder strips were equilibrated for 60 min, including washes with fresh buffer every 15 min. After stabilization, the strips were pre-contracted three times with 50 mM KCl, followed by 20 min of washout. Thereafter, the strips were again equilibrated with KH buffer and re-adjusted to passive tension of 10 mN. Subsequently, bladder contractions were induced by KCl (80 mM) in rat or carbachol in rat and humans (100 nM–10 μM, in tenfold concentration steps). When a maximal contraction level was reached, bladder strips were washed twice for 15 min with KH buffer and incubated in the presence or absence of the BK_Ca_ channel inhibitor iberiotoxin (final concentration 100 nM in 0.01 % bovine serum albumin (BSA); *w*/*v*) or the Rho-kinase inhibitor Y27,632 (final concentration 1 μM) for 30 min. Subsequently, a second contraction was induced with 80 mM KCl or a concentration of carbachol titrated to reach approximately 50 % of the original maximum response (about 1.2 and 2 μM in the groups with and without iberiotoxin or Y27,632, respectively). Cumulative concentration-response curves (1 nM–100 μM) were generated for relaxation by the nonselective β-AR agonist isoprenaline or the β_3_-AR agonist mirabegron. At the end of the experiment, 10 μM forskolin was added to define maximum relaxation.

Compared to protocols previously used to study functional mechanisms in urinary bladder (Frazier et al. 2005), our current experimental design first assessed potential effects of iberiotoxin and Y27,632 on the initial contractile stimuli and then set the obtained maximum response to 100 % to study the effects of the β-AR agonists.

In some experiments, a passive tension of 5, 10 or 15 mN was applied to human strips which were left to equilibrate in KH solution for 60 min, including washes with fresh buffer every 15 min. After stabilization, the strips were pre-contracted three times with 50 mM KCl, followed by 20 min of washout. Thereafter, the strips were again equilibrated with KH buffer and re-adjusted to passive tension of 5, 10 or 15 mN. Thereafter, the strips were relaxed by isoprenaline or mirabegron in a concentration-dependent manner.

### MLC phosphorylation studies

To determine MLC phosphorylation, we modified the above protocol as follows: after 30-min pre-incubation with and without Y27,632 (1 μM) under a passive tension of 10 mN, bladder strips were either snap-frozen immediately, after contraction with carbachol (either added as 1 μM or as tenfold concentration increments from 100 nM to 10 μM) or after carbachol followed by a β-AR agonist concentration-response curve. Some strips also were frozen 30 min after the final carbachol addition.

Frozen strips were processed for Western blot analysis of phosphorylated MLC (p-MLC) levels. Strips were pulverized under liquid nitrogen, followed by sonification in homogenization buffer (composition in mM: 50 Tris-HCl, 150.0 NaCl, 1.0 EDTA, 1.0 PMSF, 1.0 NA_3_VO_4_, 1.0 NaF, pH 7.4, supplemented with 10 μg mL^−1^ leupeptin, 10 μg mL^−1^ aprotinin, 10 μg mL^−1^ pepstatin, 0.25 % NA-deoxycholate and 1 % Igepal (NP-40)). The homogenate was centrifuged at 8800*g* for 10 min, and the supernatant was taken. The total protein concentration was determined according to Bradford (1976). Each sample containing 60 μg of total protein was dissolved in 4× Laemmli buffer (Laemmli 1970), boiled for 5 min at 95 °C, separated by 12 % sodium dodecyl sulphate-polyacrylamide gel electrophoresis (SDS-PAGE) and transferred to nitrocellulose membranes. Immunoblots were blocked with 3 % BSA in Tris-buffered saline (TBS) containing 0.1 % Tween 20 (TTBS) for 2 h at room temperature. Subsequently, they were incubated overnight at 4 °C with TTBS with 3 % BSA containing the primary antibody p-MYL9 (sc-12896; Santa Cruz Biotechnology, Santa Cruz, CA, USA) at a dilution 1:200. Bands were visualized after 1.5 h of incubation with horseradish-peroxidase-conjugated donkey anti-goat (antibody 705-035-003; Jackson ImmunoResearch; dilution 1:3000) or donkey anti-mouse secondary antibody (715-035-150; Jackson ImmunoResearch; dilution 1:3000) in TTBS with 3 % BSA, respectively, followed by chemiluminescent imaging (PerkinElmer Inc., Waltman, MA, USA). Immunoblots were analyzed by densitometry using TotalLab software (Nonlinear Dynamics, Newcastle, UK). All band intensities were normalized to GAPDH expression (antibody sc-47724; Santa Cruz Biotechnology; dilution 1:2000).

### Data analysis

The current protocol differs from that used in our previous studies with rat and human bladder strips (Frazier et al. 2011). The main points of our current data analysis are as follows: Force of contraction was expressed as the percentage of peak force in response to 80 mM KCl or 1 μM carbachol as measured prior to addition of inhibitor. For analysis of β-AR agonist effects, 0 % relaxation was defined as the force measured immediately prior to adding the first agonist concentration, and 100 % was defined as the force measured after addition of 10 μM forskolin; in experiments not involving forskolin, 100 % relaxation was defined as a tension of 10 mN. As the concentration-response curves for the β-AR agonists were shallow and/or did not reach a clear maximum response in some cases, no formal analysis of EC_50_ or maximum response was performed. Rather, the curves in the absence and presence of iberiotoxin or Y27,632 were compared by two-way ANOVA testing for effect of treatment and of agonist concentration. Differences in contractile responses or in MLC phosphorylation were assessed using paired Student’s *t* test. All data represent means ± SD from *n* experiments. The pre-defined null hypothesis in all statistical tests was that the inhibitor did not affect the response under investigation. A *p* < 0.05 was considered statistically significant.

### Chemicals

Mirabegron (also known as YM178) was provided by Astellas (Tokyo, Japan). (−)-Isoprenaline hydrochloride was obtained from Sigma (St. Louis, MO, USA). Rho-kinase inhibitor Y27,632 ((R)-(+)-trans-4-(1-aminoethyl)-*N*-(4-pyridyl)cyclohexanecarboxamide dihydrochloride) was obtained from Santa Cruz Biotechnology, iberiotoxin from Tebu-Bio (Le Perray-en-Yvelines, France) and forskolin from LC Laboratories (Woburn, MA, USA). Mirabegron and forskolin were dissolved in dimethyl sulfoxide at a concentration of 20 mM, yielding final solvent concentrations of 0.5 and 0.15 %, respectively, in the organ bath. All other compounds were dissolved in distilled water.

## Results

### Organ bath contraction studies

**Fig. 1:**
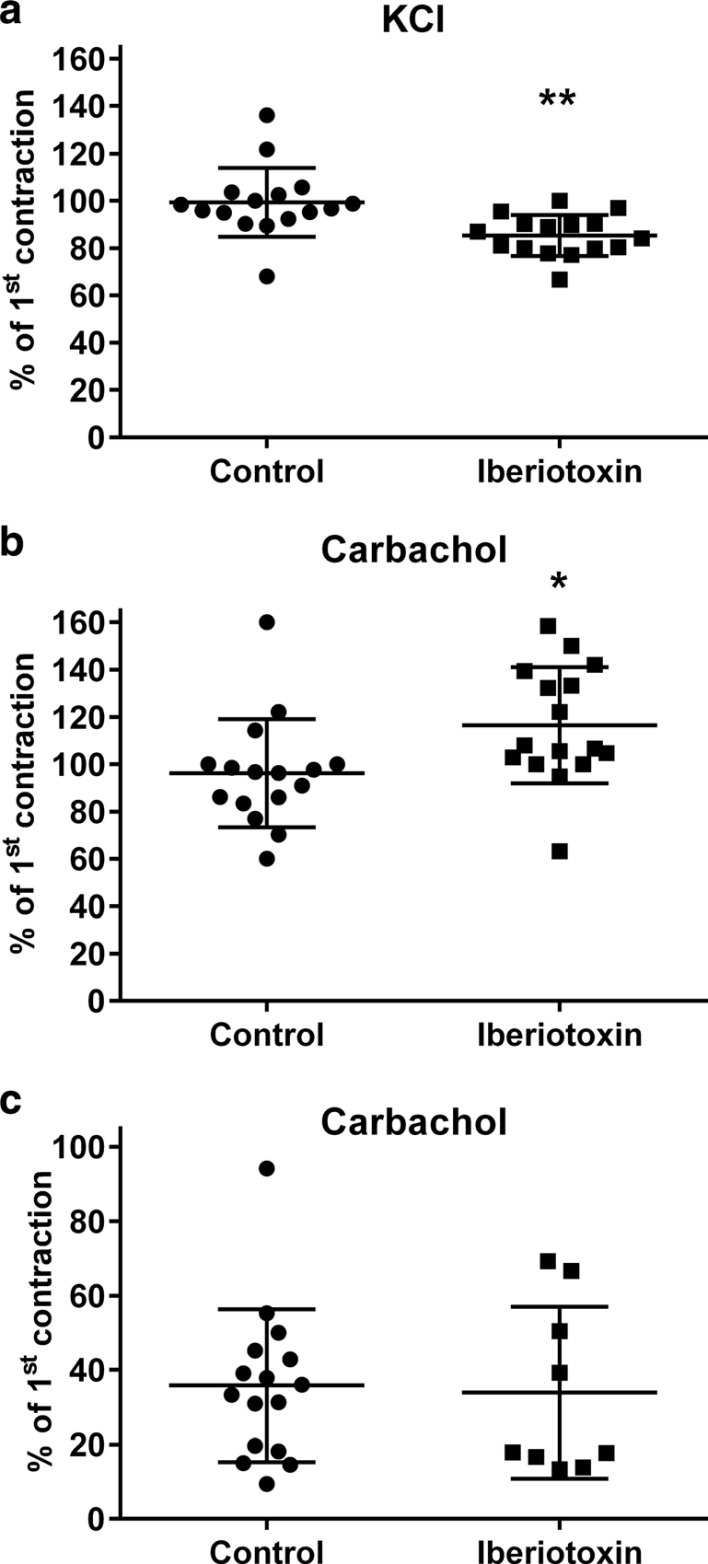
Contraction of rat bladder strips induced by 80 mM KCl (**a**) or rat (**b**) and human bladder strips (**c**) by 1 μM carbachol in the absence (control) or presence of 100 nM iberiotoxin. Data are expressed as % of the first contraction, i.e. prior to inhibitor addition, and are mean ± SD of 9–16 strips per group, *p < 0.05 and **p < 0.01 vs. control in a two-tailed Student’s *t* test

**Fig. 2:**
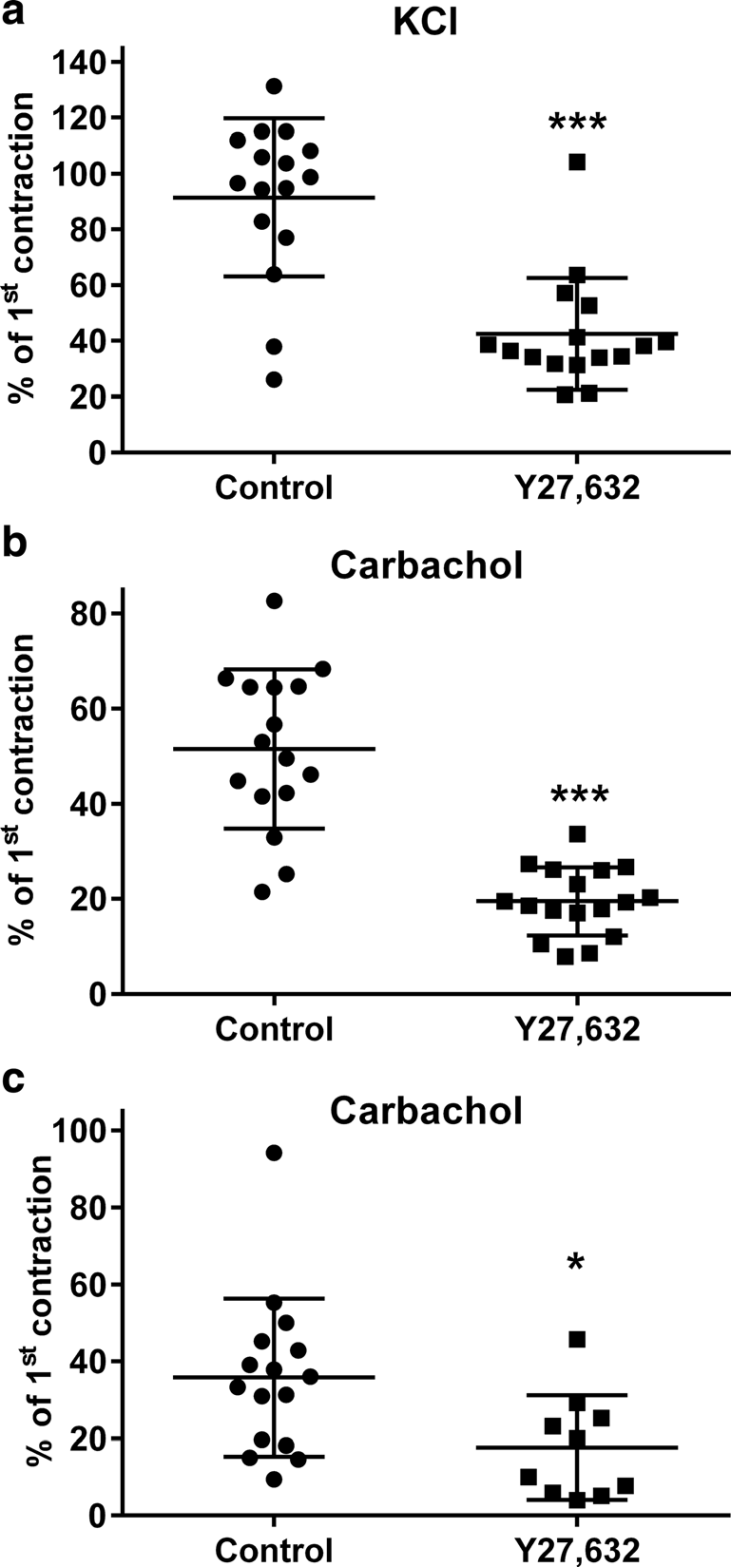
Contraction of rat bladder strips induced by 80 mM KCl (**a**) or rat (**b**) and human bladder strips (**c**) by 1 μM carbachol in the absence (control) or presence of 1 μM Y27,632. Data are expressed as % of the first contraction, i.e. prior to inhibitor addition, and are mean ± SD of 10–17 strips per group, *p < 0.05 and ***p < 0.001 vs. control in a two-tailed Student’s *t* test

The initial contraction response of rat urinary bladder strips to 80 mM KCl and to 1 μM carbachol, i.e. before addition of any inhibitor, was 25.9 ± 11.3 and 33.2 ± 20.9 mN, respectively (based on 64 and 62 strips, respectively). All subsequent contraction data are presented as % of this initial response. The KCl-induced contraction in rat was slightly reduced (*p* < 0.05) by the BK_Ca_ channel inhibitor iberiotoxin (100 nM; Fig. 1a). In contrast, iberiotoxin slightly enhanced (*p* < 0.05) the contractile response to 1 μM carbachol (Fig. 1b), indicating that the two contractile stimuli may use different signalling pathways to induce contraction. In rat bladder strips, the Rho-kinase inhibitor Y27,632 (1 μM) similarly decreased KCl- and carbachol-induced contraction by about 60 % (*p* < 0.05) (Fig. 2a, b).

The degree of passive tension (5, 10 and 15 mN) in human bladder strips had no major effect on the relaxation responses to isoprenaline or mirabegron (Suppl. Fig. 1). The initial contraction response of human bladder strips to 1 μM carbachol, i.e. before addition of any inhibitor, was 21.2 ± 12.5 mN (*n* = 35 strips); all subsequent contraction data are presented as % of this initial response. Iberiotoxin did not affect the contractile response to 1 μM carbachol (Fig. 1c), whereas Y27,632 reduced carbachol-induced contraction by half (*p* < 0.05) (Fig. 2c) in human bladder.

### Organ bath relaxation studies

**Fig. 3:**
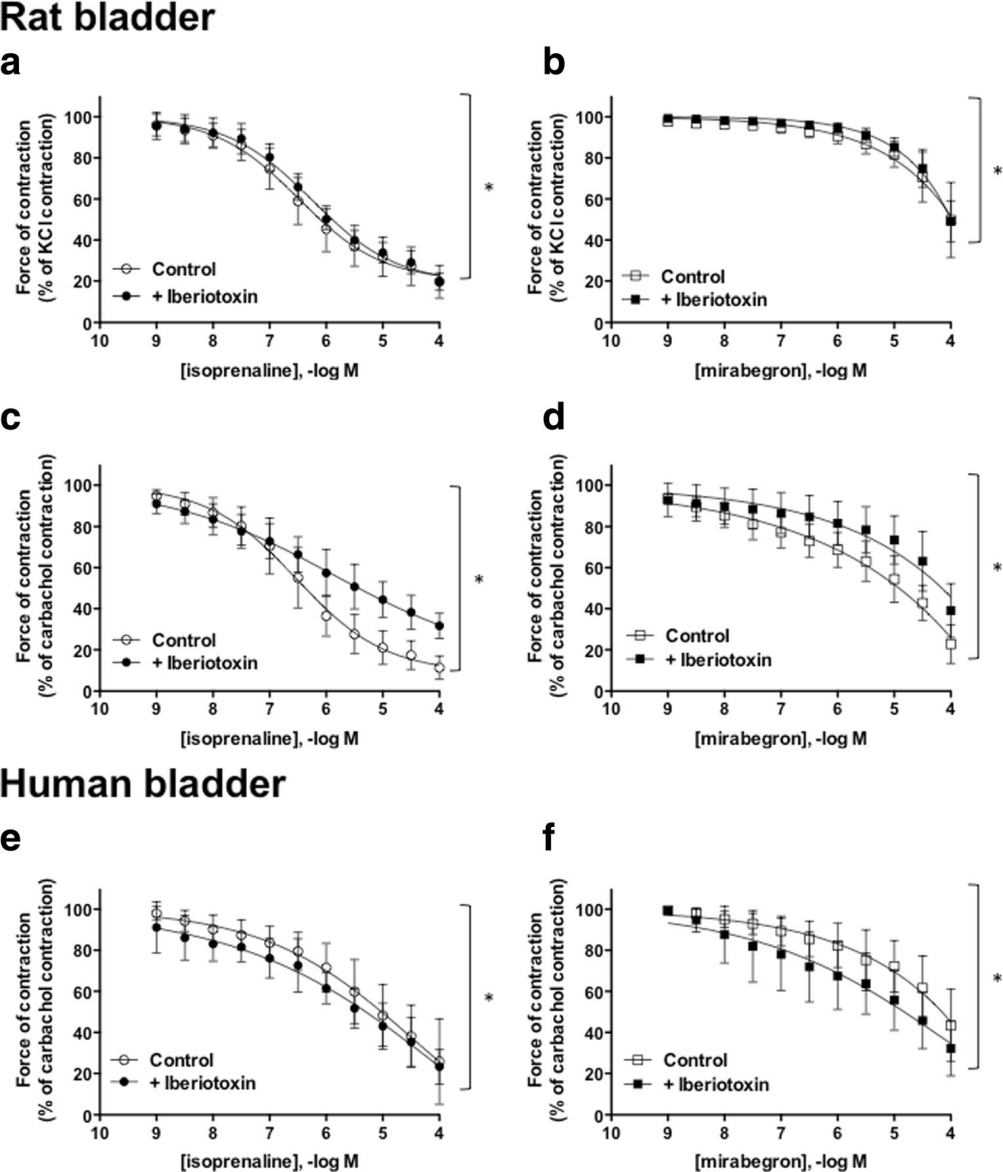
Relaxation of rat (**a**–**d**) and human (**e**, **f**) bladder strips pre-contracted by 80 mM KCl (**a**, **b**) or 1 μM carbachol (**c**–**f**) with isoprenaline (*left panels*) or mirabegron (*right panels*) in the absence (*open symbols*) or presence (*filled symbols*) of 100 nM iberiotoxin. Data are expressed as % of tension measured immediately prior to β-AR agonist addition and are mean ± SD (*n* = 8–9 per group). In a two-way analysis of variance, the effect of iberiotoxin was *p* < 0.05 in all six panels

**Fig. 4:**
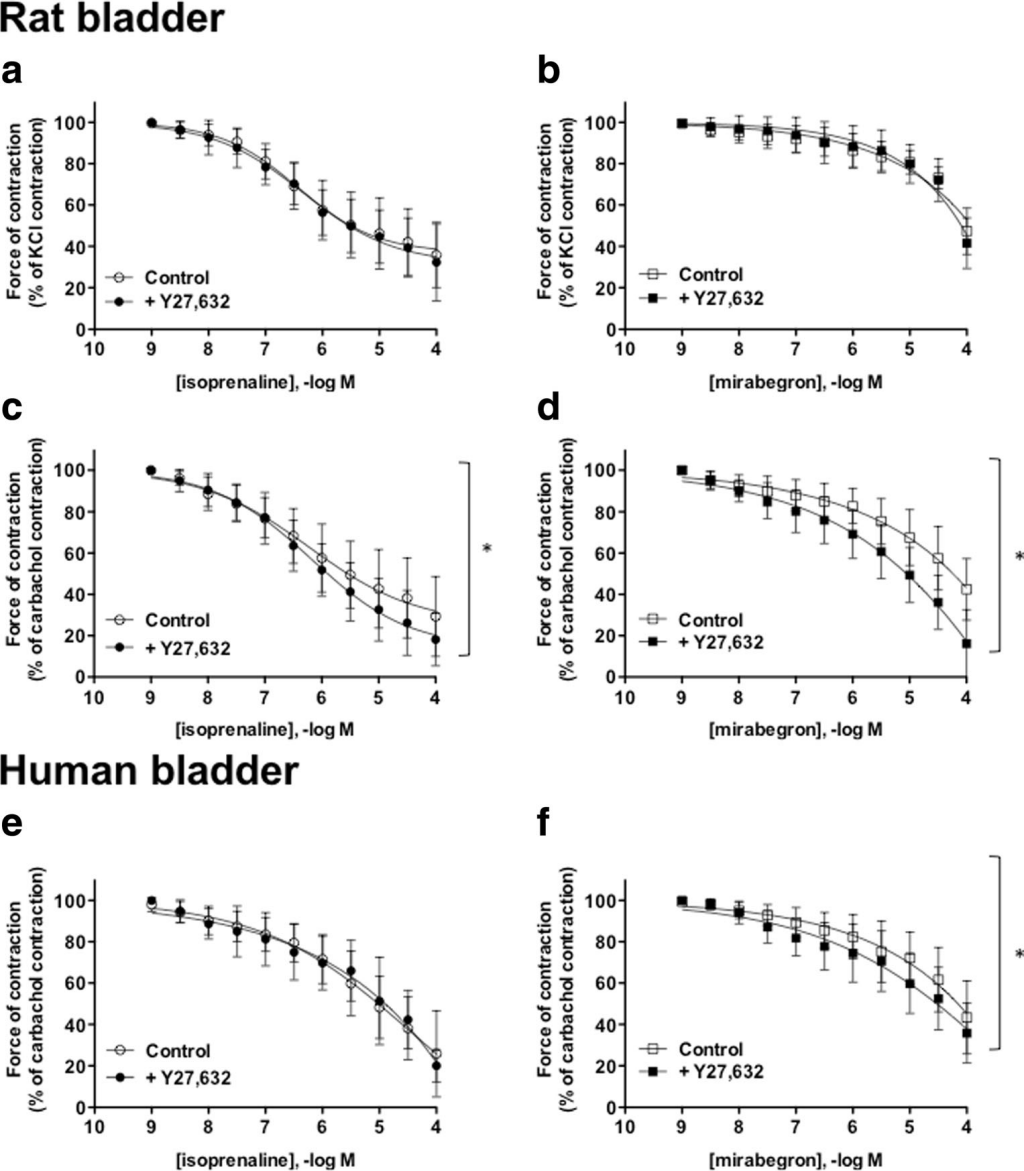
Relaxation of rat (**a**–**d**) and human (**e**, **f**) bladder strips pre-contracted by 80 mM KCl (**a**, **b**) or 1 μM carbachol (**c**–**f**) with isoprenaline (*left panels*) or mirabegron (*right panels*) in the absence (*open symbols*) or presence (*filled symbols*) of 1 μM Y27,632. Data are expressed as % of tension measured immediately prior to β-AR agonist addition and are mean ± SD (*n* = 6–9 per group). In a two-way analysis of variance, the effect of Y27,632 was *p* < 0.05 when relaxation was tested against carbachol in the rat for both agonists and in humans with mirabegron

The general β-AR agonist isoprenaline caused rat bladder relaxation and, in this regard, appeared less potent against carbachol- than against KCl-induced tone (Figs. 3a, c and 4a, c). The β_3_-selective agonist mirabegron was less potent in inducing rat bladder relaxation than isoprenaline and, within the tested concentration range, yielded somewhat smaller maximum relaxation, especially up to 10 μM (Figs. 3b, d and 4b, d).

In rat bladder, iberiotoxin only slightly attenuated relaxation in response to either isoprenaline or mirabegron against KCl-induced tone (Fig. 3a, b) but attenuated the relaxation response to both agonists against carbachol-induced tone to a larger degree (Fig. 3c, d).

The presence of Y27,632 did not affect relaxation responses to either isoprenaline or mirabegron against KCl-induced tone (Fig. 4a, b) but enhanced the relaxation response to both agonists against carbachol-induced tone (Fig. 4c, d).

In contrast to rat bladder, isoprenaline and mirabegron had comparable potency for relaxation of carbachol-induced tone in human bladder (Figs. 3e, f and 4e, f). Also, in contrast to rat bladder, iberiotoxin slightly enhanced the effects of both agonists (Fig. 3e, f). Y27,632 did not alter isoprenaline-induced relaxation but slightly enhanced that by mirabegron (Fig. 4e, f) in human bladder.

### Rat bladder MLC phosphorylation studies

The bladder strips used to assess MLC phosphorylation were distinct from those used in the above contraction and relaxation studies, and slightly different experimental conditions were applied, i.e. shorter protocol, use of passive tension, 1 and 10 μM carbachol and lack of forskolin at the end of the experiment. In these relaxation experiments, we found that Y27,632 slightly reduced the relaxation response of isoprenaline against passive tension but not that of mirabegron (Suppl. Fig. 2a). Y27,632 did not affect the decline of carbachol-induced tension over time (Suppl. Fig. 2b). The potency of both isoprenaline and mirabegron to induce relaxation was somewhat greater against 1 μM than against 10 μM carbachol-induced tone, but the effects of Y27,632 on such relaxation were comparable to the data presented in Fig. 4 (Suppl. Fig. 2c, d).

**Fig. 5:**
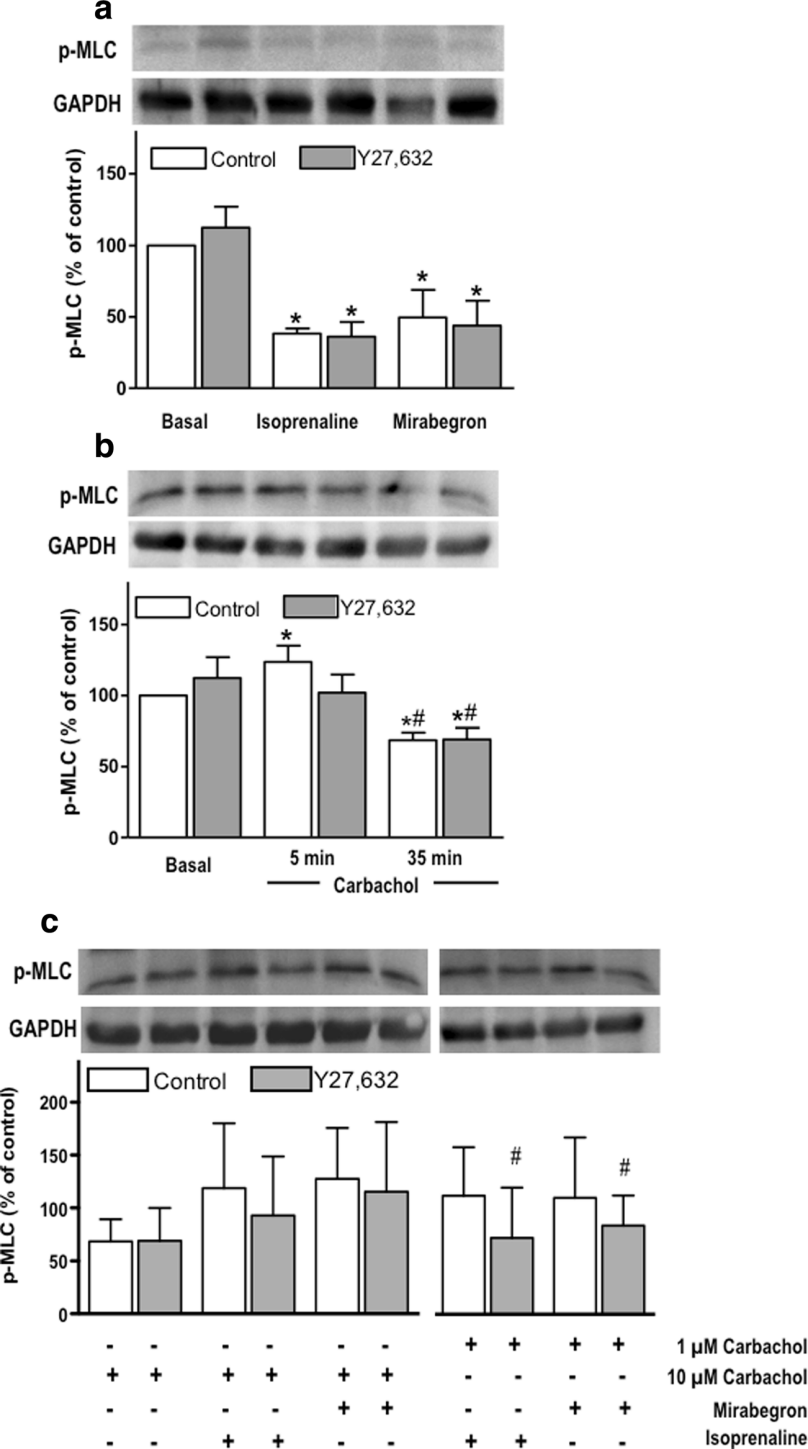
Myosin light chain (MLC) phosphorylation in rat bladder strips in response to isoprenaline and mirabegron after pre-contraction with carbachol or passive tension in the presence (*grey bars*) or absence (*empty bars*) of 1 μM Y27,632. **a** MLC phosphorylation in bladder strips under passive tension of 10 mN (basal) with following stimulation in concentration-dependent manner by isoprenaline or mirabegron. **b** Phosphorylation of MLC strips under passive tension (basal) and strips pre-contracted with 10 μM carbachol for 5 and 35 min. **c** All subsequent MLC phosphorylation data were obtained at the 35-min time point. Rat bladder strips were either pre-contracted by 10 μM carbachol with following relaxation induced by isoprenaline and mirabegron or pre-contracted with 1 μM carbachol and relaxed by adding isoprenaline and mirabegron in concentration-dependent manner. Equal loading was verified by analysis of GAPDH. Representative blots and densitometric quantification of *n* = 4–13 independent experiments are shown. Data are expressed as percentage of phosphorylated MLC in control group ± SD, * < 0.05 vs. basal; # < 0.05 vs. 10 μM carbachol in an unpaired, two-tailed Student’s *t* test

The degree of MLC phosphorylation in bladder strips with a passive tension of 10 mN was markedly reduced by both isoprenaline and mirabegron, whereas Y27,632 affected neither the basal values nor the reductions by the two agonists (Fig. 5a). Exposure to 10 μM carbachol for 5 min slightly increased MLC phosphorylation, whereas exposure for 35 min reduced it; while the minor increase at 5 min was not detected in the presence of Y27,632, the inhibitor had no effect on basal values or those after 35 min of carbachol (Fig. 5b). All subsequent MLC phosphorylation data were obtained at the 35-min time point. In the presence of 10 μM carbachol, both isoprenaline and mirabegron numerically increased MLC phosphorylation, but this did not reach statistical significance (Fig. 5c). The extent of MLC phosphorylation with isoprenaline or mirabegron in the presence of 1 μM carbachol was similar to that in the presence of 10 μM carbachol (Fig. 5c). Y27,632 numerically reduced MLC phosphorylation in the presence of either β-AR agonist, but this only reached statistical significance in the presence of 1 μM carbachol (Fig. 5c).

## Discussion

### Critique of methods

In our current study, we used urinary bladder tissue from both rats and humans. Relaxation in rat is mediated by a combination of β_2_- and β_3_-ARs but in humans is predominantly, if not exclusively, by β_3_-AR (Michel and Vrydag 2006; Igawa et al. 2012). Therefore, we have used the prototypical agonist isoprenaline, which similarly activates all β-AR subtypes, and mirabegron, which is a β_3_-selective agonist (Igawa and Michel 2013).

The Rho-kinase inhibitor Y27,632 concentration-dependently reduces contractile bladder responses to many stimuli including muscarinic agonists (Schneider et al. 2005), bradykinin (Sand and Michel 2014) and KCl (Rajasekaran et al. 2007). In the present study, we have used a concentration of Y27,632 which had inhibited contraction only by about 20 % in previous studies (Schneider et al. 2005). While this is likely to underestimate effects of this Rho-kinase inhibitor, higher concentrations may have affected contractile tone by a degree that is unsuitable for relaxation experiments. Moreover, higher concentrations of Y27,632 might inhibit other kinases such as protein kinase C (Davies et al. 2000). Such approach most likely preserves a contraction level sensitive to relaxation but implies a chance for underestimating a contribution of Rho-kinase.

Several studies have demonstrated that β_3_-AR agonists relax human bladder at resting tension (Biers et al. 2006; Igawa et al. 2012). In our present study, these observations were expanded to different degrees of pre-tension in human bladder. Here, we show that both β-AR agonists were similarly potent in causing relaxation against a pre-tension of 5, 10 and 15 mN.

Finally, as, under pathological conditions, acetylcholine release also occurs during the storage phase of the micturition cycle (Yoshida et al. 2009), we focused on the role of iberiotoxin and Y27,632 in relaxation mediated by β-AR agonists against carbachol-induced contraction in both rat and human urinary bladder.

Nevertheless, the K^+^ concentration of urine in urinary bladder is also known to fluctuate, and as lower concentrations of KCl have already been studied (Frazier et al. 2005; Uchida et al. 2005), we investigated the effect of pre-contraction with 80 mM KCl in rats. This might indeed underestimate the role of BK_Ca_ channels in a relaxation, but in agreement with previous studies, we confirmed the role of BK_Ca_ channels in a relaxation against even high concentration of K^+^.

### Role of BK_Ca_ channels

BK_Ca_ channels are critical regulators of detrusor smooth muscle excitability and contractility; specifically, the activation of BK_Ca_ channels is functionally linked to β-AR signalling involved in the relaxation of detrusor smooth muscle (Frazier et al. 2008; Petkov 2014). We initially tested the effects of the BK_Ca_ channel inhibitor iberiotoxin on bladder contraction. This yielded conflicting results depending on experimental conditions. Thus, in agreement with a study performed in rabbit small intestine (Lamarca et al. 2006), iberiotoxin reduced 80 mM KCl-induced contraction in rat detrusor smooth muscle. On the other hand, iberiotoxin slightly enhanced carbachol-induced contraction in rat bladder. In isolated whole neonatal rat bladder, iberiotoxin enhanced the carbachol effect (Ng et al. 2006), and a recent study showed that activation of M_3_ receptors with carbachol induced transient BK_Ca_ channel activation in isolated rat urinary smooth muscle cells, and this phenomenon was eliminated upon inhibiting inositol-3-phosphate receptors with xestospongin C (Parajuli and Petkov 2013). On the other hand, no iberiotoxin effect against carbachol was observed in human bladder in the present study. Lack of iberiotoxin effect on carbachol-induced pre-contraction in humans could be most likely a consequence of a reduction of maximum response to carbachol-induced contraction in the control group which occurs in consecutive carbachol-induced contractions (Schneider et al. 2004a).

Previous studies on the effect of iberiotoxin or other BK_Ca_ channel inhibitors in rat bladder have been performed with relaxation against passive tension or against contraction induced by 40–50 mM KCl (Frazier et al. 2005; Uchida et al. 2005). While the absence or presence of KCl did not affect the ability of isoprenaline to concentration-dependently elevate cAMP formation, iberiotoxin inhibited relaxation by isoprenaline or the β_3_-AR agonist FR165101 against KCl-induced contraction but not against passive tension. In the present study, qualitatively similar but quantitatively smaller inhibition of relaxation was observed with the β-AR agonists isoprenaline and mirabegron. The smaller extent of iberiotoxin effect most likely reflects the higher KCl concentration that we have used (80 vs. 40–50 mM), as the extent of effects of BK_Ca_ channel ligands in the bladder generally depends on extracellular K^+^ concentration (Trivedi et al. 1995). Iberiotoxin produced greater attenuation of isoprenaline and mirabegron responses in rat bladder against carbachol, a condition not previously explored by other investigators; these were quantitatively similar to those seen in rat bladder when contraction had been induced by 40–50 mM KCl (Frazier et al. 2005; Uchida et al. 2005). However, in human bladder strips, iberiotoxin had opposite effects and enhanced relaxation induced by both isoprenaline and mirabegron. In contrast, iberiotoxin had attenuated relaxation against field stimulation-induced contraction in human bladder, a response which is mediated by a combination of muscarinic and purinergic mechanisms (Afeli et al. 2013). Similar to the effects on contraction, we find it difficult to determine whether these differences between contraction protocol and species reflect biological differences or data variability. Given the effect sizes of iberiotoxin in all of these studies and despite “statistical significance” within each study, we cannot fully exclude the possibility that some of these differences represent random variation.

### Role of Rho-kinase

In agreement with previous studies reporting on the critical role of Rho-kinase in bladder contraction (Schneider et al. 2005; Rajasekaran et al. 2007), the Rho-kinase inhibitor Y27,632 reduced contraction induced by both KCl and carbachol in rat, as well as carbachol-induced contraction in human detrusor smooth muscle. Despite the known role of Rho-kinase in regulation of bladder tone (Peters et al. 2006), its role in β-AR-induced relaxation has not been studied previously. Here, we provide evidence that Rho-kinase involvement in β-AR downstream signalling seems to depend on the contractile agonist; this is not surprising as receptor-dependent and receptor-independent agonists use different signalling pathways to cause bladder contraction. Y27,632 did not change isoprenaline- or mirabegron-induced relaxation against KCl-induced contraction in rat, indicating that β-AR-mediated relaxation against KCl-induced pre-contraction does not depend on Rho-kinase inhibition. The role of Rho-kinase in β-AR-mediated relaxation upon pre-contraction by carbachol, however, remains unclear; on the one hand, Y27,632 enhanced relaxation mediated by both isoprenaline and mirabegron in rats and by mirabegron in humans; on the other hand, neither agonist reduced MLC phosphorylation in the presence of carbachol.

To study a potential role of Rho-kinase-dependent mechanism underlying β-agonist-induced relaxation after pre-contraction with carbachol, we measured levels of MLC phosphorylation. Here, we show that against passive tension, both β-AR agonists induced relaxation along with a decrease in MLC phosphorylation, although the extent of change differed between the two responses. In contrast, in carbachol pre-contracted strips, relaxation by β-AR agonists was not followed by reduced levels of MLC phosphorylation. In addition, Y27,632 did not influence MLC phosphorylation despite improved functional relaxation against passive tension or carbachol pre-contraction. Only after pre-contraction with 1 μM carbachol, Y27,632 reduced MLC phosphorylation in bladder strips relaxed by both isoprenaline and mirabegron.

In agreement with the hypothesis that stretch of detrusor muscle is able to regulate smooth muscle tone upon enhanced basal MLC phosphorylation (Ratz and Miner 2003), we found increased levels of MLC phosphorylation in bladder strips under tension of 10 mN. Although Rho-kinase has been shown to be constitutively active in bladder smooth muscle (Poley et al. 2008), Y27,632 did not influence the increased MLC phosphorylation. As expected, we report here that the carbachol-induced MLC phosphorylation time dependently decreased even reaching levels below basal. Inhibition of Rho-kinase prevented carbachol-induced MLC phosphorylation, but Y27,632 did not alter MLC phosphorylation over time. The later observation was confirmed at the functional level. Our finding that both β-AR agonists were more potent against 1 μM than against 10 μM carbachol-induced contraction in rat bladder does not necessarily contradict these findings. The higher concentration of carbachol produces more pronounced muscarinic receptor stimulation, known to affect the potency of a β-AR agonist (Longhurst and Levendusky 1999; Michel and Sand 2009; Witte et al. 2011).

Surprisingly, addition of β-AR agonists increased MLC phosphorylation in the presence of carbachol while mediating relaxation at the functional level. Y27,632 significantly decreased MLC phosphorylation only in the presence of 1 μM carbachol-induced pre-contraction. β_3_-ARs couple to adenylyl cyclase stimulation, but the functional role of cAMP in the bladder remains unclear (Frazier et al. 2005; Uchida et al. 2005). Our results indicate that reduction of carbachol-induced contraction by β-AR agonists might be mediated by other signalling pathways, which are activated in a carbachol-concentration-dependent manner.

In summary, we confirmed a contribution of BK_Ca_ channels in β-AR-mediated relaxation against pre-contraction using 80 mM KCl in rat, although this was smaller than against that reported with lower KCl concentrations; we extended such findings to relaxation induced by β-AR agonists after pre-contraction with carbachol in rat and human urinary bladder. We demonstrated that Rho-kinase inhibition enhanced relaxation induced by the β_3_-selective agonist mirabegron upon carbachol-induced contraction in both rat and human. The latter finding was not accompanied by decreased MLC phosphorylation, but MLC phosphorylation induced by carbachol decreased in the presence of Y27,632. Collectively, our study indicates that the signalling pathway involved in relaxation induced by β-AR agonists at least partly depends on the contractile stimulus; this had been suspected before based on the differential potency and/or efficacy of β-AR to cause relaxation against tone induced by carbachol vs. that induced by other agonists in bladder, airways and other tissues (for review see Dale et al. 2014). In the absence of a contractile stimulus, β-AR agonists inhibit MLC phosphorylation. In contrast, reduction of carbachol-induced contraction seems to be mediated by other signalling pathways, the latter being activated in carbachol-concentration-dependent manner. Overall, it seems that unidentified pathways influence KCl- and carbachol-induced contractions and β-AR-mediated relaxation in urinary bladder.

## Electronic supplementary material

ESM 1 (DOC 4975 kb)
