# Supplementary material for: β3-Adrenoceptor-mediated relaxation of rat and human urinary bladder: roles of BKCa channels and Rho kinase
Source: Naunyn Schmiedebergs Arch Pharmacol. 2015 May 9;388(7):749–59. doi: 10.1007/s00210-015-1128-z (PMC4475246; doi:10.1007/s00210-015-1128-z)
Supplement: Supplementary file 1 — (DOC 4975 kb) [file 210_2015_1128_MOESM1_ESM.doc]

Online supplement to

**β_3_-Adrenoceptor-mediated relaxation of rat and human urinary bladder: roles of BK_Ca_ channels and Rho kinase**

Hana Cernecka^1a^, Kim Kersten^1^, Harm Maarsingh^1^, Carolina R. Elzinga^1^, Igle Jan de Jong^2^, Cees Korstanje^3^, Martin C. Michel^4^, Martina Schmidt^1^

SUPPLEMENTAL FIGURES

Supplemental figure 1: Effects of different levels of resting tone (5, 10 and 15 mN) on isoprenaline and mirabegron-induced relaxation in human detrusor strips. Data are expressed as mean ± SD of 5 independent experiments per group. In a two-way analysis of variance the effect of pre-contraction was *p<0.05 when relaxation was tested against all three different degrees of pre-tension for mirabegron and #p<0.05 for isoprenaline when tested 5 mN vs. 15 mN.

Supplemental figure 2: (A) Development of rat urinary bladder tone in the presence of isoprenaline and mirabegron after treatment with or without 1 µM Y27,632 under conditions of passive tension. (B) Strips pre-contracted with 10 µM carbachol were left to maintain sustained phase of contraction (Time Control) in the presence or absence of 1 µM Y27,632. Relaxation of rat bladder strips pre-contracted by either 1 µM or 10 µM carbachol upon addition of indicated concentrations of isoprenaline (C) or mirabegron (D) measured in the presence or absence of 1 µM Y27,632. Data are expressed as mean ± SD of 7-8 (A), 16 (B) and 7-8 (C, D) per group. In a two-way analysis of variance the effect of Y27,632 was *p<0.05 when relaxation was tested against passive tension in the rat for isoprenaline (A), against passive tension (B), and against 1 µM carbachol for both agonists (C, D).
